# Editorial

**Published:** 1872-06

**Authors:** 


### Editorial.

### *Rush Medical College.*

Rush College enters upon a new era, and, in so doing, justifies the confidence which it has heretofore enjoyed, and merits a success which shall surpass the eminent prosperity which has characterized its past history.

The destruction of the magnificent structure erected in the summer of 1867, was a loss which seemed irreparable; but at the present time events indicate that the loss, great as it was, will result in a close connection between the College and Cook County Hospital, and thus achieve an end which would not have been realized in the old location.

The plans of the Trustees relative to rebuilding upon the old site have been completely changed by the determination of the County Commissioners to permanently locate the County Hospital. The building heretofore occupied for hospital purposes is owned by the city, and is located upon leased grounds. The overcrowded state of the wards forces upon the commissioners the work of providing further accommodation, and they are about to select a new location. The uncertainty as to the time when this change would be made, which existed five years since, prevented the Trustees of the College from attempting to establish a proximity to the Hospital. Now, however, so near at hand is this important step that the Trustees have concluded to erect *temporary* lecture and dissecting rooms for the accommodation of the class during the ensuing session. By the kindness of the commissioners, these rooms are erected upon the hospital grounds, and when the Hospital is moved, Rush College will move with it.

The announcement, which is just issued, contains the following:

"The advantages of such a connection with the largest hospital in the West, affords facilities to the students of Rush College, which will far more than compensate the great loss sustained by the destruction of our former splendid edifice.

"While the lecture and dissection rooms, which are now in process of erection, are of a temporary nature, the fire ordinance compels the use of brick in their construction, and the Faculty, under whose direction the structure is being erected, have provided ample accommodations in both rooms. Large in size, free in ventilation, and thoroughly warmed, the lectures being so arranged as to frequently change the location of the class from the operating theatre of the Hospital to the lecture-room of the College, every consideration of health and comfort will be fully realized.

"The lecture-room will seat, comfortably, over three hundred students, each seat being numbered, as in the old College. This plan enables the student, by sending to the Treasurer of the Faculty the matriculation fee in advance of the Session, to secure a desirable seat, and forestalls the rush for seats which characterizes the ingress of the class to the lecture-room, in colleges where this system does not prevail.

"The Trustees and Faculty consider that the permanent proximity to the County Hospital, which will hereafter characterize Rush Medical College, and the requirements of the College for graduation, fully comply with the spirit of the age, and the demand of the profession for practical training of medical students. Cook County Hospital must ever be the largest hospital in Chicago, and the municipal character of the charity will necessarily furnish the greatest variety of disease and accident. During the last lecture session there were daily clinics, and during the ensuing term the same system will prevail."

